# ARTEMIS stabilizes the genome and modulates proliferative responses in multipotent mesenchymal cells

**DOI:** 10.1186/1741-7007-8-132

**Published:** 2010-10-27

**Authors:** Sarah A Maas, Nina M Donghia, Kathleen Tompkins, Oded Foreman, Kevin D Mills

**Affiliations:** 1 The Jackson Laboratory, 600 Main Street, Bar Harbor ME 04609, USA; 2 Adnexus Therapeutics, Waltham, MA 02453, USA; 3 Division of Infectious Diseases, University of North Carolina, Chapel Hill, NC27599, USA; 4 The Jackson Laboratory, 4910 Raley Road, Sacramento CA, 95838, USA; 5 Main Medical Center Research Institute, Scarborough Maine, USA

## Abstract

**Background:**

Unrepaired DNA double-stranded breaks (DSBs) cause chromosomal rearrangements, loss of genetic information, neoplastic transformation or cell death. The nonhomologous end joining (NHEJ) pathway, catalyzing sequence-independent direct rejoining of DSBs, is a crucial mechanism for repairing both stochastically occurring and developmentally programmed DSBs. In lymphocytes, NHEJ is critical for both development and genome stability. NHEJ defects lead to severe combined immunodeficiency (SCID) and lymphoid cancer predisposition in both mice and humans. While NHEJ has been thoroughly investigated in lymphocytes, the importance of NHEJ in other cell types, especially with regard to tumor suppression, is less well documented. We previously reported evidence that the NHEJ pathway functions to suppress a range of nonlymphoid tumor types, including various classes of sarcomas, by unknown mechanisms.

**Results:**

Here we investigate roles for the NHEJ factor ARTEMIS in multipotent mesenchymal stem/progenitor cells (MSCs), as putative sarcomagenic cells of origin. We demonstrate a key role for ARTEMIS in sarcoma suppression in a sensitized mouse tumor model. In this context, we found that ARTEMIS deficiency led to chromosomal damage but, paradoxically, enhanced resistance and proliferative potential in primary MSCs subjected to various stresses. Gene expression analysis revealed abnormally regulated stress response, cell proliferation, and signal transduction pathways in ARTEMIS-defective MSCs. Finally, we identified candidate regulatory genes that may, in part, mediate a stress-resistant, hyperproliferative phenotype in preneoplastic ARTEMIS-deficient MSCs.

**Conclusions:**

Our discoveries suggest that *Art *prevents genome damage and restrains proliferation in MSCs exposed to various stress stimuli. We propose that deficiency leads to a preneoplastic state in primary MSCs and is associated with aberrant proliferative control and cellular stress resistance. Thus, our data reveal surprising new roles for ARTEMIS and the NHEJ pathway in normal MSC function and fitness relevant to tumor suppression in mesenchymal tissues.

## Background

Nonhomologous end joining (NHEJ) is a critical DNA double-stranded break repair pathway, important in the repair of general DNA double-stranded breaks and programmed DSBs generated during B- and T-lymphocyte development [1,2]. Cells lacking NHEJ exhibit variable proliferative defects, hypersensitivity to ionizing radiation and other clastogens and spontaneous chromosomal instability. Numerous studies have implicated NHEJ as a key suppressor of lymphoid tumorigenesis, both in humans and in experimental models [3-5]. The tumor-suppressive role of NHEJ is thought to be largely via prevention of oncogenic chromosomal rearrangements relating to failed lymphocyte development. More recently, we and others have shown that NHEJ is a tumor-suppressive pathway in multiple nonlymphoid tissues, though the detailed mechanisms remain essentially unknown [6-8].

ARTEMIS (encoded by the *Art/Dclre1c *gene) is a DNA processing exo/endonuclease that acts together with the DNA-dependent protein kinase (DNA-PK) to prepare DNA ends for ligation by the core NHEJ machinery [9,10]. Accumulating evidence has also pointed to DNA damage response or checkpoint activation roles for *Art *that may be distinct from its DNA repair activities [11-15]. Thus, *Art *may uniquely function as a key integrator of DNA damage signals, cellular response and DNA repair. In this context, *Art *is important in both general DNA double-stranded break (DSB) repair and in specialized repair of programmed DNA breaks during V(D)J recombination in developing B- and T-lymphocytes [3,9,10]. Mutations in *Art *underlie human radiosensitive severe combined immunodeficiency (RS-SCID) and SCID-A, primary immunodeficiencies associated with hypersensitivity to DNA-damaging agents and variable lymphoma predisposition [5,9,10,16-18].

We recently demonstrated a role for *Art *in suppression of several classes of sarcomas, including osteosarcomas, chondrosarcomas, rhabdomyosarcomas and poorly differentiated anaplastic sarcomas. In this context, we postulate a role for *Art *in suppressing neoplastic transformation of mesenchymal stem/progenitor cells (MSCs), closely related multipotent mesenchymal stromal cells (MMS), or their descendants. MSCs and MMSs are multipotent cells that can give rise to multiple lineages, including bone, cartilage, fat and muscle [19-23]. MSCs may also be capable of hepatic, renal, cardiac or neural differentiation, at least in some limited contexts. In the bone marrow, MSCs and MMSs function as a stem/progenitor cell reservoir for renewal/replacement of numerous skeletal or associated cell types and function as stromal cells supporting hematopoietic stem cell differentiation/development [20,23-25]. Owing to their capacity to differentiate along many different axes, MSCs are an extremely attractive candidate for use in regenerative medicine applications [20,24]. In this context, it is critical to understand the mechanisms that govern both normal MSC fitness and activity and potential pathologies, especially cancers that may be linked with MSC derangement. However, the factors that influence the normal tissue-regenerative functions, while preventing neoplastic transformation, of MSCs remain poorly understood.

Our previous data suggested that patients with mutations in *Art *or other NHEJ factors may also be at risk for a host of nonlymphoid cancers, especially sarcomas, even if the immunodeficiency can be corrected by bone marrow transplantation or gene therapy [8]. Here we have further investigated the mechanistic role of ARTEMIS in mesenchymal tumor suppression and in normal MSC function and fitness. The importance of ARTEMIS in primary MSC derived from the bone marrow microenvironment was tested in detail using knockout mice [26]. We find that ARTEMIS is important for normal proliferative control of MSCs, especially after exposure to various cytostress stimuli. These findings add to the growing evidence that, in addition to DNA repair functions, ARTEMIS is a key factor in normal cell cycle response to cellular stressors, such as DNA damage [11-14]. In this context, we propose that *Art*-deficient MSCs acquire a preneoplastic state in which normal proliferative control is altered. The relevance to sarcomagenesis is discussed.

## Results and Discussion

### The NHEJ factor ARTEMIS suppresses sarcomagenesis

**Figure 1:**
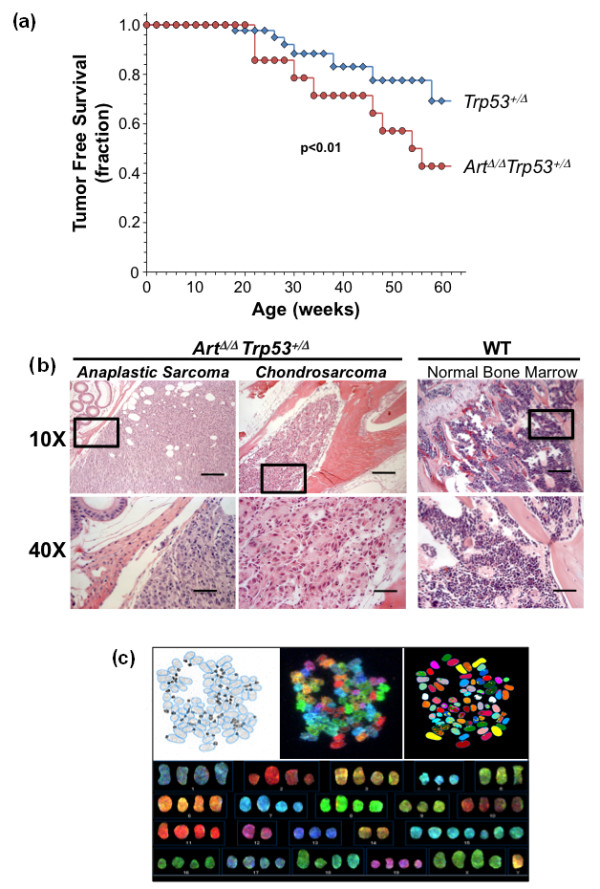
***Art *deficiency accelerates mesenchymal tumor development**. **(a) **Tumor-free survival analyses of *Art*^Δ/Δ ^*Trp53*^Δ/+ ^mice (red circles) versus *Trp53*^Δ/+ ^mice (blue, diamond). Plotted is the surviving fraction of those mice that developed tumors as a function of time (weeks). Significance was determined by *t*-testing. **(b) **Hematoxylin and eosin (H&E) staining of an anaplastic sarcoma (left) and a chondrosarcoma (center) found in *Art*^Δ/Δ^*/Trp53*^Δ/+ ^mice, shown at ×10 and ×40 magnification. Normal bone marrow from an *Art *mouse (right) is shown for comparison. Boxed areas in ×10 magnification demarcate regions shown in ×40 magnification. Lines represent scale bars (200 μm in ×10 magnification; 50 μm in ×40 magnification). **(c) **Spectral karyotype (SKY) analysis of *Art*^Δ/Δ ^*Trp53*^+/Δ ^osteosarcoma. Shown are the 4',6'-diamidino-2-phenylindole (DAPI) stained metaphase (inverted image, top left) with superimposed chromosome contours (blue), spectral image of SKY painted metaphase spread (top, middle), and computer classified image (top, right), as well as the karyotype table showing approximate hyperdiploidy (bottom).

In the context of a lymphoma study, we previously observed that deficiency for ARTEMIS in mice can be associated with increased incidence of certain nonlymphoid tumors, including sarcomas. Because *Trp53*^Δ/+ ^mice are predisposed to broad-spectrum tumorigenesis, including various sarcomas, we reasoned that effects of *Art *deficiency would be readily detectable on the tumor-sensitized *Trp53*-heterozygous background [27-29]. To investigate the broad, tumor-suppressive functions of ARTEMIS, we generated, in total, 750 *Art*-knockout (*Art*^Δ/Δ^) mice that were heterozygous for the p53 tumor suppressor gene (*Trp53*). Of the 750 *Art*^Δ/Δ ^*Trp53*^Δ/+ ^mice, 46 (6.1%) developed tumors of any kind. This is similar to, but slightly lower than, the overall tumor incidence (17%) previously reported for *Art*^Δ/Δ ^*Trp53*^Δ/+ ^mice [30]. Of the 46 mice developing tumors in our cohort, 14 (30.4%) developed sarcomas of various subtypes. This is roughly the same as the overall sarcoma incidence previously described for *Trp53*^Δ/+ ^[27,29]. Whereas the fraction of mice ultimately developing sarcomas was similar in our *Art*^Δ/Δ ^*Trp53*^Δ/+ ^and *Trp53*^Δ/+ ^control mice, a higher fraction of *Art*^Δ/Δ ^*Trp53*^Δ/+ ^animals developed sarcomas over the initial 60-week observation period (Figure 1a). This suggests that *Art *deficiency may accelerate tumorigenesis in *Trp53 *heterozygote animals. Histopathological analysis after hematoxylin and eosin (H&E) staining revealed a range of sarcoma subtypes occurring in *Art*^Δ/Δ ^*Trp53*^Δ/+^, including chondrosarcoma, osteosarcoma and rhabdomyosarcoma (Figure 1b). Notably, anaplastic sarcomas with poor, ambiguous or heterogeneous differentiation were also consistently observed (Figure 1b). Taken together, these data imply a role for ARTEMIS in suppressing a range of tumors, including several sarcoma subtypes in a tumor-sensitized *Trp53*^Δ/+ ^context.

Although we could only obtain metaphase chromosome spreads from two *Art-*deficient sarcomas, spectral karyotype analysis of this sarcoma subset revealed aneuploidy and hyperdiploidy, without grossly detectable chromosomal translocations (Figure 1c; Additional file 1). While we cannot rule out chromosomal instability in some *Art*-deficient sarcomas, the spectral karyotypes we did analyze were reminiscent of human sarcoma karyotypes, which commonly show aneuploidization without consistent or clonal translocations [31-41]. These data suggest that *Art *may suppress sarcomagenesis by mechanisms that are at least partly independent of chromosomal stability control. In this context, the observed aneuploidization may be indicative of a defect in normal proliferation or growth control in the pretransformed or early transformed sarcoma cells of origin.

### *Art *is required for genome stability in primary MSCs

The occurrence of poorly differentiated sarcomas in *Art*^Δ/Δ ^*Trp53*^Δ/+ ^mice, as well as the range of differentiated cell types identified, suggested origination of these diverse tumors from a common precursor cell type. We therefore focused on the role of ARTEMIS in multipotent primary MSCs as candidate sarcoma cells of origin. We first evaluated whether *Art *is expressed in normal MSCs by a reverse transcriptase polymerase chain reaction (RT-PCR) assay, originally used to analyze *Art*-knockout (*Art*^Δ/Δ^) embryonic stem cells [42]. Total RNA was isolated from either wild-type (WT) or *Art*^Δ/Δ ^MSCs, isolated by standard methods from corresponding mice, and *Art *transcript was measured by RT-PCR [19,42,43]. This verified that *Art *is transcriptionally expressed in WT MSCs and confirmed ablation of *Art *in mutant MSCs (Additional file 2).

**Figure 2:**
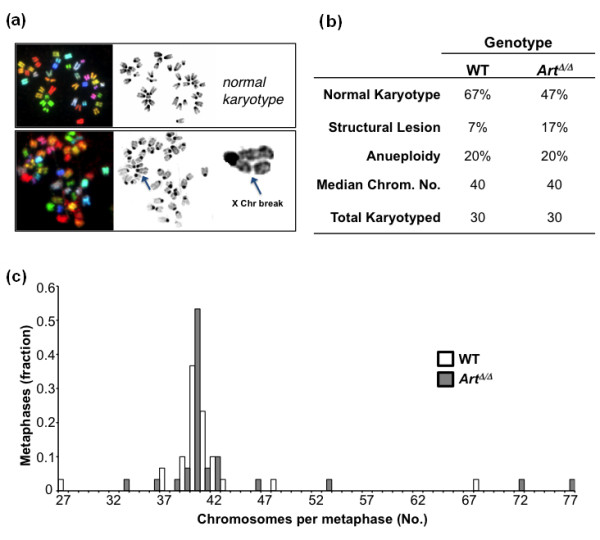
***Art *prevents chromosome instability in MSCs**. **(a) **SKY analysis of wild type (WT) (top) or *Art*^Δ/Δ ^(bottom) mesenchymal stem cells (MSCs). An example of a chromatid break, typifying damage in *Art*^Δ/Δ ^cells, is indicated by an arrow and shown magnified. **(b) **Summary of spontaneous chromosomal abnormalities in WT versus *Art*^Δ/Δ^. **(c) **Distribution of chromosome number in WT versus *Art*^Δ/Δ ^MSC karyotypes. Shown is the percentage of metaphase spreads from each karyotype harboring the indicated number of chromosomes.

We next assessed whether *Art *acts to prevent spontaneous chromosomal instability in MSCs. Untreated WT or *Art*^Δ/Δ ^primary MSCs were analyzed by conventional or spectral karyotyping (SKY). This revealed approximately the same rate (20%) of spontaneously occurring aneuploidy in *Art*^Δ/Δ ^and WT MSCs (Figures 2a-c). More detailed analysis of *Art*^Δ/Δ ^versus WT MSC metaphase spreads revealed similar overall range, distribution and median in number of chromosomes per cell (Figures 2b and 2c). However, *Art*^Δ/Δ ^MSCs also exhibited a higher frequency of spontaneous chromosomal structural lesions, that is, breaks, fragments, or translocations, than their WT counterparts (17% versus 7%, respectively) (Figures 2a and 2b). Collectively, these data suggest that *Art *is critical to maintain overall genome stability in primary MSCs, with key roles in preventing chromosome fragmentation and aneuploidy. However, these functions may be unrelated, or indirectly related, to sarcoma suppression functions, as we find evidence for sarcomagenesis without translocations.

### *Art *is dispensable for MSC differentiation

**Figure 3:**
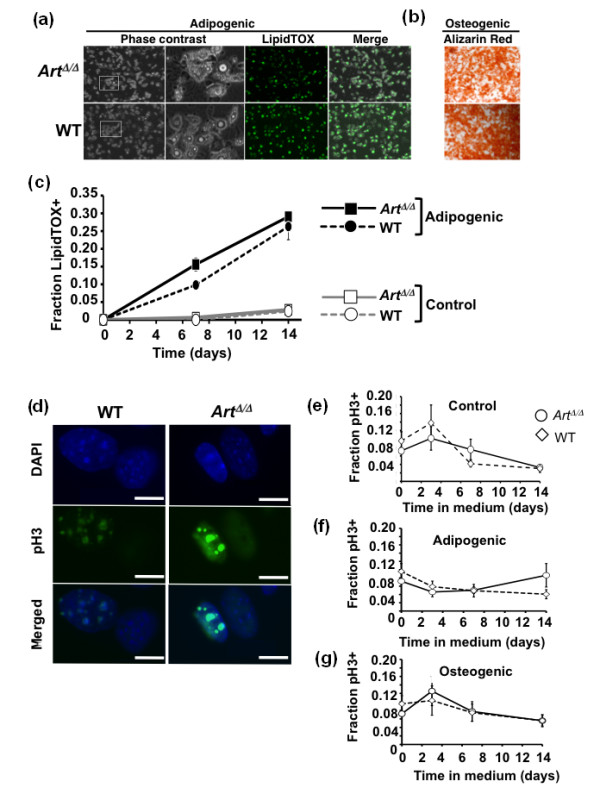
***Art*-deficient MSCs differentiate normally**. **(a) ***Art*^Δ/Δ ^and WT MSCs were grown in adipogenic culture medium for 7 days, fixed and stained with the fluorescent lipid-binding dye LipidTOX (Invitrogen). Shown are bright field, fluorescent and merged images for each. **(b) ***Art*^Δ/Δ ^and WT MSCs were grown in osteogenic culture medium for 14 days, fixed and stained with Alizarin red to detect mineralization indicative of osteocytic development. (c) *Art*^Δ/Δ ^and WT MSCs were grown in adipogenic or unsupplemented culture medium for 14 days, fixed and stained with LipidTOX and DAPI counterstain. The fraction of LipidTOX-positive cells was determined for each sample and culture condition at days 0, 7 and 14. Error bars indicate standard error. **(d-f) **Mitotic indices for undifferentiated, adipogenic or osteogenic cultures of *Art*^Δ/Δ ^and WT MSCs were determined by immunostaining for M-phase marker phosphorylated histone H3 (phospho-H3). **(d) **Representative fluorescence micrographs of phospho-H3-positive cells (green), DAPI DNA counterstain (blue), and merged are shown for *Art*^Δ/Δ ^and WT MSCs. Scale bars, 10 μm. Fractions of positive phospho-H3 staining were determined at days 0, 3, 7 and 14 of **(e) **undifferentiated control, **(f) **adipogenic or **(g) **osteogenic culture conditions. Error bars indicate standard error.

We next tested whether *Art *deficiency affected MSC differentiation competency. Normal MSCs can differentiate into multiple cell types, including lipid-producing adipocytes and calcium-depositing osteocytes [21-23]. For these analyses, WT and *Art*^Δ/Δ ^MSCs were therefore cultured under adipogenic or osteogenic, as well as nondifferentiating (control), conditions (Figure 3) [19]. Cellular response to differentiation medium was first tested by evaluating changes in gross cell morphology via light microscopy (Figure 3a). Responses to adipogenic medium were apparent as early as 3 days after induction for both WT and *Art*^Δ/Δ ^cultures, with cells evincing a rounded morphology and accumulating characteristic lipid droplets. After 7 days of adipogenesis, a substantial fraction of both WT and *Art*^Δ/Δ ^cultures contained large lipid vacuoles (Figure 3a). To confirm adipogenic differentiation, WT or *Art*^Δ/Δ ^MSC cultures were fixed and stained with the lipid binding fluorescent dye LipidTOX (Invitrogen, Carlsbad, CA, USA) (Figure 3a). WT and *Art*^Δ/Δ ^MSC cultures each contained a high percentage of fluorescently labeled cells that were qualitatively indistinguishable from one another (Figure 3a). Similarly, *Art*^Δ/Δ ^MSCs cultured under osteogenic conditions exhibited morphological changes and calcium deposition comparable to WT MSCs, indicating essentially normal osteoid differentiation potential (Figure 3b). As controls for differentiation specificity, *Art*^Δ/Δ ^and WT control MSC cultures were stained for off-target differentiation (Additional file 3). Neither *Art*^Δ/Δ ^nor control cultures showed evidence of inappropriate differentiation. Together, these data demonstrate that *Art*^Δ/Δ ^MSC retain grossly normal differentiation potential. To functionally evaluate primary MSC recovery from bone marrow preparations and to quantify differentiation competency, *Art*^Δ/Δ ^versus WT MSC, LipidTOX-positive cells were measured as a function of the total cell count at 0, 7 or 14 days after transfer to adipogenic, or nondifferentiating, culture conditions (Figure 3c). Neither *Art*^Δ/Δ ^nor WT MSC cultures showed significant increases in the LipdTOX-positive fraction under nondifferentiating conditions up to 14 days of culture (Figure 3c, gray lines). Conversely, both *Art*^Δ/Δ ^and WT exhibited nearly identical increases in LipidTOX positivity at both 7 and 14 days of adipogenic culture (Figure 3c, black lines). These results demonstrate that *Art *deficiency does not significantly affect either the number or the differentiation competency of primary MSCs relative to WT.

In addition to general differentiation competency, MSCs were assessed for proliferative responses during differentiation. WT or *Art*^Δ/Δ ^MSCs were transferred from standard growth to either adipogenic, osteogenic or control medium, cultured for up to 14 days and measured at various culture time points for mitotic index as a marker of actively proliferating cells by immunostaining for phosphorylated histone H3 (phospho-H3), a mitosis-specific marker.

During culture in nondifferentiation, adipogenic or osteogenic medium *Art*^Δ/Δ ^and WT MSC cultures exhibited similar phospho-H3 cell counts throughout the 14-day measurement time course (Figures 3d-g). Together with the differentiation data above, these results demonstrate that *Art *deficiency does not quantitatively affect the number of bone marrow MSCs, as defined by functional differentiation and cell proliferation assays.

### *Art *modulates the response to induced DNA damage in MSCs

**Figure 4:**
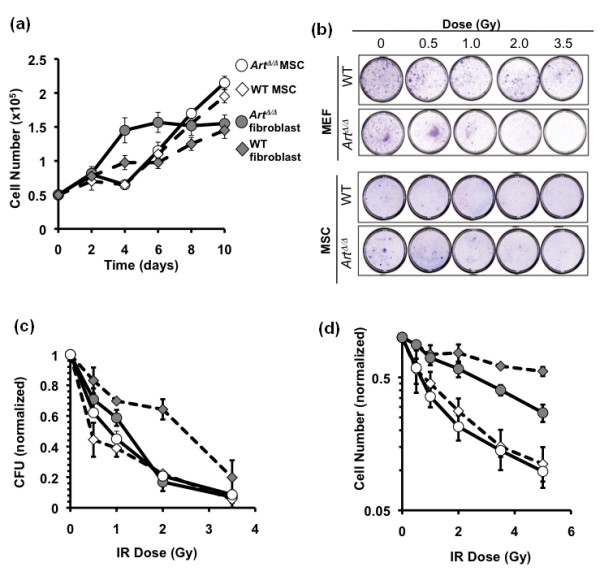
***Art *is dispensable for MSC resistance to ionizing irradiation**. **(a) **Three independent biological replicates of WT or *Art*^Δ/Δ ^MSCs or control fibroblasts were plated at 5 × 10^4 ^cells per dish each and cultured for 10 days. Viable cell counts, measured by exclusion of the vital dye trypan blue, were determined every 2 days. Error bars indicate standard error. **(b) **Clonogenicity assay for sensitivity to ionizing irradiation. A total of 1 × 10^5 ^WT or *Art*^Δ/Δ ^MEFs or 5 × 10^5 ^WT or *Art*^Δ/Δ ^MSCs were irradiated at the indicated doses, plated to 100-mm dishes and cultured until colonies were visibly evident for the unirradiated WT controls of each cell type. Cells on all plates were then fixed and stained with crystal violet histological stain. **(c) **Quantification of clonogenicity (from **(b)**) following IR. Colonies were counted and normalized to the unirradiated control for MEFs or MSCs, respectively. Error bars indicate standard error. **(d) **WT and *Art*^Δ/Δ ^MSCs or control fibroblasts were exposed to ionizing radiation (IR) at indicated doses, plated at equivalent densities in triplicate and harvested for analysis after 7 days. Viable cell counts were determined for single-cell suspensions by trypan blue exclusion. Relative resistance to IR is expressed as the viable cell count at each dose normalized to the unirradiated control for each sample. Error bars denote standard error.

Because the NHEJ pathway is critical in other cell types for resistance to DNA double-stranded break-inducing agents such as ionizing radiation (IR), we asked whether *Art*^Δ/Δ ^MSCs were radiosensitive relative to WT controls. Initially, growth rates for WT versus *Art*^Δ/Δ ^MSCs were compared in the absence of irradiation. Low-passage isolates of primary WT or *Art*^Δ/Δ ^MSCs or mouse embryonic fibroblast (MEF) controls were seeded in replicate cultures with fresh medium at the same density (5 × 10^4 ^total cells), and expansion was assessed by manual cell counting at 2-day intervals for 10 days (Figure 4a). This analysis revealed no overt differences between *Art*^Δ/Δ ^and WT MSCs or MEFs, although the *Art*^Δ/Δ ^MEFs showed a slightly faster initial expansion than their counterpart WT MEFs (Figure 4a). Overall, this indicated that *Art *deficiency did not grossly compromise MSC growth under nonstress conditions.

Next, we evaluated *Art*^Δ/Δ ^versus WT MSCs for radiosensitivity by two different assays. First, we performed a colony formation assay following irradiation of WT or *Art*^Δ/Δ ^MSCs at doses ranging from 0 to 3.5 Gy. After irradiation, serial dilutions were plated and cultured until colonies were visibly evident in unirradiated samples. Colony counts were determined after staining with crystal violet, and all data were normalized to unirradiated colony counts. As controls, radiosensitivity was also determined for *Art*^Δ/Δ ^or WT fibroblasts, with the former previously shown to be hypersensitive to IR exposure [26]. *Art*^Δ/Δ ^fibroblasts expectedly showed IR hypersensitivity relative to WT fibroblasts, especially at intermediate doses (Figure 4c). By contrast, *Art*^Δ/Δ ^and WT MSCs were essentially indistinguishable for radiosensitvity at all doses tested, but MSCs of both genotypes were overall significantly more IR-sensitive than WT fibroblasts (Figure 4c). As a second assay, we measured the total number of viable cells in culture, irrespective of colony-forming potential, after irradiation at doses ranging from 0 to 5 Gy. Cell suspensions of *Art*^Δ/Δ ^or WT MSCs or MEFs were irradiated, plated and cultured for 7 days, and then scored for viable cells by manual cytometry. Surviving cell counts were normalized to unirradiated cultures (Figure 4d). As in the colony formation assay, we observed the relative IR hypersensitivity in *Art*^Δ/Δ ^MEFs, but not MSCs, relative to the corresponding WT cells. Notably, by this assay, both WT and *Art*^Δ/Δ ^MSCs were more sensitive than their counterpart MEFs to IR at all doses.

### *Art *modulates MSC proliferation following ionizing irradiation

**Figure 5:**
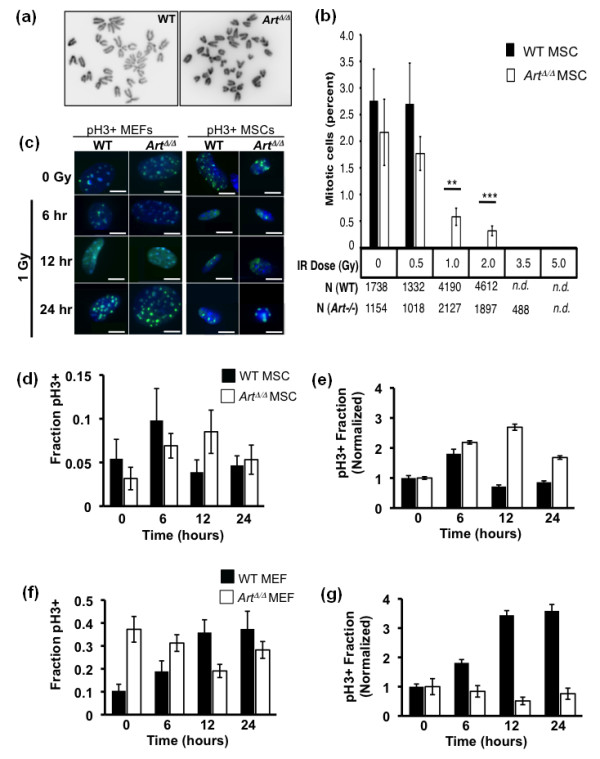
***Art *modulates cell cycle response following IR**. **(a)** Representative micrographs of WT and *Art*^Δ/Δ ^MSC metaphase spreads. **(b) **The mitotic indices of WT (filled bars) and *Art*^Δ/Δ ^(open bars) MSCs following exposure to IR at indicated doses were determined by quantification of metaphase cells. Mitotic index is expressed as the percentage of mitotic figures per total nuclei. Significance was determined by *t*-testing (**P < 0.01; ***P < 0.005). **(c-g) **The mitotic indices of WT and *Art*^Δ/Δ ^MSCs or control fibroblast 6, 12 and 24 hours following 1 Gy ionizing irradiation were determined by immunostaining for the mitotic marker pH3. **(c) **Shown are representative merged micrographs of pH3+ (green) and DAPI DNA counterstained (blue) WT or *Art*^Δ/Δ ^MEFs and MSCs at each time point after IR. Scale bars, 10 μm. The fraction of phospho-H3-positive cells for WT (filled bar) and *Art*^Δ/Δ ^(open bar) MSCs **(d) **or MEFs **(f) **were determined for each time point after IR. Data from **(d **and **f) **were also normalized to the 0 Gy controls. Normalized data are shown for MSC **(e) **and MEF **(g)**. Error bars indicate standard error.

At sublethal doses, clastogens such as IR can also provoke either temporary or permanent arrest of mitotic activity in normal cells. To test whether *Art *modulates MSC proliferative control following irradiation, *Art*^Δ/Δ ^or WT control MSCs were γ-irradiated and cultured as described above, but mitotic activity was determined by measuring the fraction of metaphase nuclei after 24 hours of recovery (Figures 5a and 5B). *Art*^Δ/Δ ^MSC cultures showed slightly lower mitotic indices than WT cultures at 0 and 0.5 Gy, but significantly higher mitotic indices at 1 and 2 Gy (Figures 5a and 5b). Above 2 Gy, mitotic activity in both genotypes was dramatically reduced, and cellular viability of WT MSC was significantly impaired (Figure 5b).

To measure the kinetics of mitotic response to IR, WT or *Art*^Δ/Δ ^MSCs were subjected to 1 Gy of γ-irradiation, and the mitotic index was determined by immunofluorescent detection of phosphorylated histone H3 (pH3) at 0, 6, 12 or 24 hours after irradiation (Figures 5c-g). As controls, WT or *Art*^Δ/Δ ^MEFs were similarly irradiated and analyzed, with the latter known to be hypersensitive to IR exposure. WT MSCs showed an initial increase in mitotic index following irradiation, approximately doubling by 6 hours, then declining to preirradiation levels by 12 hours (Figures 5d and 5e). Relative to WT, *Art*^Δ/Δ ^MSCs showed a prolonged mitotic response to IR, with mitotic index increasing twofold by 6 hours, reaching a peak level of 2.5-fold by 12 hours and remaining elevated at 24 hours (Figures 5d and 5e). By contrast, *Art*^Δ/Δ ^MEFs showed elevated mitotic index in unirradiated cultures relative to WT MEFs, but did not exhibit a sustained increase in mitotic index following irradiation as in WT cells (Figures 5f and 5g).

Altogether, these results suggest that *Art *is dispensable for overall resistance to genotoxic stress in multipotent adult MSCs, but that *Art *critically modulates MSC cell cycle response following ionizing irradiation. The underlying basis for the dichotomy between MSCs and fibroblasts for IR resistance is not known, but a similar phenomenon was previously observed in the context of embryonic stem cells (ESCs) [26,41]. *Art *deficiency did not hypersensitize ESCs to DNA damaging agents, but WT and *Art*^Δ/Δ ^ESCs were generally more sensitive than corresponding fibroblasts. It is possible that this represents a difference in the importance of the NHEJ pathway in stem/progenitor versus more differentiated cell types.

### *Art*-deficient MSCs are resistant to serum deprivation stress

**Figure 6:**
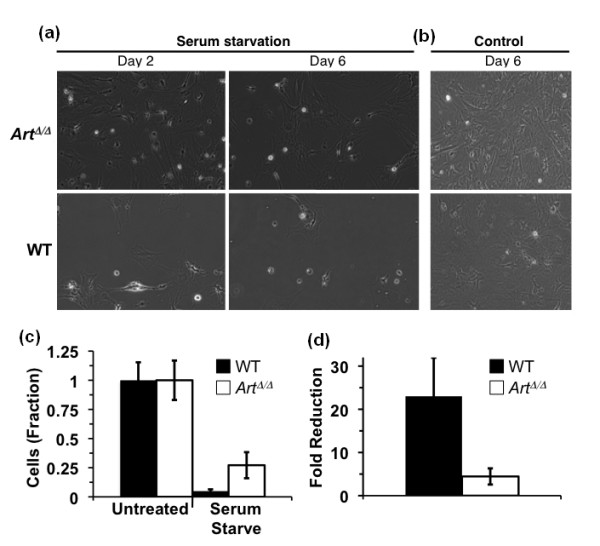
***Art*-deficient MSCs are resistant to culture stress by serum withdrawal**. **(a)** Light micrographs of *Art*^Δ/Δ ^or WT MSCs exposed to serum-free (serum starvation) versus normal (10%) serum (control) culture conditions. Cell cultures were photographed after either 2 or 6 days of serum withdrawal. **(b) ***Art*^Δ/Δ ^and WT MSCs after culture in normal conditions for 6 days. **(c) **Viability of WT (filled bars) versus *Art*^Δ/Δ ^(open bars) MSCs after culture in normal (control) or serum withdrawal (serum starved) conditions for 7 days. Viable cell counts were determined as the number of trypan blue-excluding cells normalized to the normal serum control. **(d) **Fold reduction in survival of WT (filled bars) or *Art*^Δ/Δ ^(open bars) following 7 days of serum withdrawal. Significance in all assays was determined by *t*-testing (***P < 0.005).

Previous studies have suggested that normal MSCs are acutely sensitive to culture stress, especially by diminished serum concentration [44,45]. We therefore tested whether lack of *Art *affected MSCs' sensitivity to serum deprivation. Initially, after transfer to serum-free medium, *Art*^Δ/Δ ^MSCs appeared indistinguishable from WT, for both cell density and morphology (Figure 6a). However, pronounced differences in cellular morphology and density rapidly manifested between WT and *Art*^Δ/Δ ^MSCs following serum withdrawal (Figures 6a and 6b; Additional files 4, 5, 6, 7). *Art*^Δ/Δ ^cells were remarkably resistant to serum starvation as compared with WT MSCs. After 6 days in serum-free medium, *Art*^Δ/Δ ^cultures retained adherent, viable cells with largely normal morphology (Figure 6a; Additional file 5). By contrast, WT MSC cultures exhibited a decrease in cell density accompanied by marked changes in cell morphology, including rounding and detachment from the culture substrate (Figure 6a; Additional file 7). After 7 days of serum deprivation, *Art*^Δ/Δ ^or WT MSCs were harvested and the remaining viable cells were counted (Figures 6c and 6d). This confirmed that *Art*-mutant MSC cultures were significantly more resistant to serum withdrawal than WT MSC, with *Art*^Δ/Δ ^cultures exhibiting greater than fourfold higher survival than WT cultures (Figures 6c and 6d).

### Misregulation of stress response, proliferation and differentiation pathways in *Art*^Δ/Δ ^MSCs

**Figure 7:**
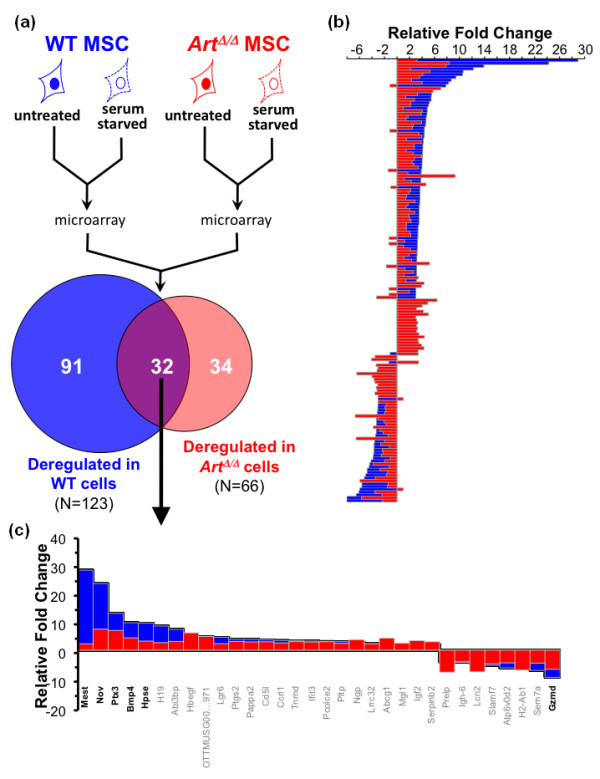
***Art*^Δ/Δ ^MSCs show an attenuated transcriptional response to serum withdrawal**. **(a) **Schematic showing experimental design for comparative gene expression analysis. RNA was isolated from duplicate cultures representing either WT (blue) or *Art*^Δ/Δ ^(red) MSCs cultured in either normal (solid line with filled nuclei) or serum starvation (dashed line with open nuclei) media. All samples were analyzed by hybridization to Affymetrix GeneChip Mouse 430 2.0 microarrays. Relative fold change (RFC) in transcription levels was determined for serum-starved cells versus corresponding controls in WT and in *Art*^Δ/Δ ^samples. Using a threshold of threefold or greater RFC, WT and *Art*^Δ/Δ ^data were comparatively analyzed and results were categorized as unique to WT cells (blue), unique to *Art*^Δ/Δ ^cells (red) or common to both (overlap). In total, 157 genes were identified with a threefold or greater RFC in WT or *Art*^Δ/Δ ^or both. Number of genes identified in each category is indicated on the Venn diagram. **(b) **RFC data for each of the 157 genes in **(a)**. Shown are RFC data for each gene in WT cells (blue bars) and in *Art*^Δ/Δ ^cells (red bars). Negative RFC values indicate lower expression in serum-starved cells relative to control; positive RFC values indicate elevated expression in serum-starved cells relative to controls. **(c) **RFC for genes common to both WT and *Art*^Δ/Δ ^cells from **(a)**. Plotted are RFC for WT (blue) and *Art*^Δ/Δ ^(red) cells as in **(b)**.

To begin identifying genetic pathways involved in the remarkable resistance of *Art*^Δ/Δ ^MSCs to serum withdrawal stress, we carried out a microarray-based comparative transcriptome analysis. Freshly isolated WT or *Art*^Δ/Δ ^MSC cultures were grown in duplicate experiments under either normal or serum withdrawal conditions (identical to above), RNA was isolated from each culture, and samples were analyzed for differential gene expression changes via hybridization to Affymetrix GeneChip Mouse Genome 430 2.0 microarrays (Affymetrix, Santa Clara, CA, USA). Gene expression differences between serum-starved and normal cells were identified independently for WT and for *Art*^Δ/Δ ^MSC cultures and expressed as a relative fold change (RFC) in the serum-starved relative to normal conditions (Figure 7a). By this approach, a total of 91 genes with a greater than threefold difference (either upregulated or downregulated) were uniquely identified for WT MSCs, while only 34 genes showed greater than threefold differences specifically in *Art*^Δ/Δ ^MSC and 32 genes were common to both WT and *Art*^Δ/Δ ^MSCs (Figures 7b and 7c). Of the 157 genes deregulated in either WT or *Art*^Δ/Δ ^MSC, the majority (109 of 157 = 69%) showed a higher RFC in WT cultures than in the corresponding *Art*^Δ/Δ ^cultures (Figure 7b). Similarly, among the deregulated genes common to both WT and *Art*^Δ/Δ ^cells, 19 (59%) of 32 exhibited a higher RFC in the WT than in the *Art*^Δ/Δ ^samples (Figure 7c). These data suggest that *Art*^Δ/Δ ^cells experience a muted overall response to serum withdrawal, manifested as a less dynamic change in gene expression relative to WT. In this context, *Art*^Δ/Δ ^MSCs are likely resistant to serum deprivation owing to an overall attenuated biological response.

**Figure 8:**
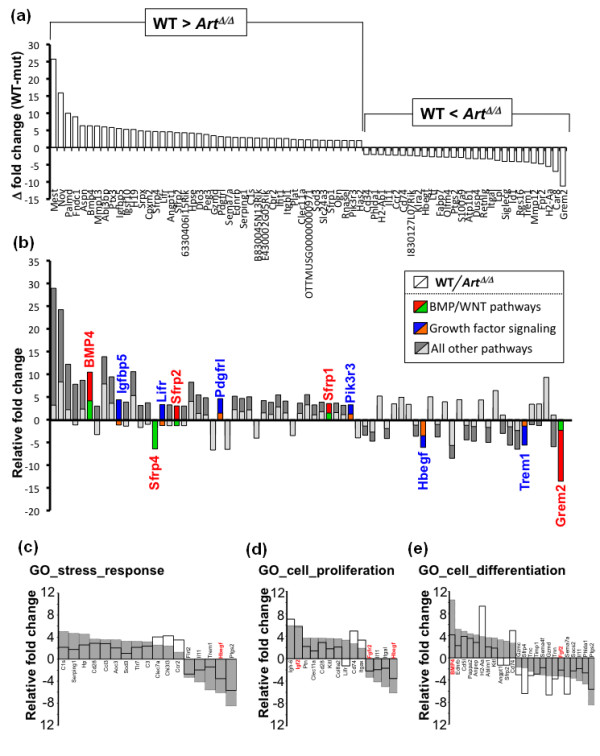
**Deregulation of stress-response, proliferation and differentiation pathways in serum-starved *Art*^Δ/Δ ^MSCs**. **(a) **Difference in RFC (ΔRFC) between WT and *Art*^Δ/Δ ^cells (defined as [WT RFC] - [*Art^Δ/Δ ^*RFC]) was determined for the 157 genes identified in Figure 7a. Plotted are data for all genes showing ΔRFC = 2 or greater. Positive ΔRFC values indicate a higher RFC in WT than in *Art*^Δ/Δ ^samples; conversely, negative ΔRFC values denote lower RFC in WT than in *Art*^Δ/Δ ^samples. Individual gene names are indicated. **(b) **RFC for genes in **(a) **are indicated, with WT (dark fill) and *Art*^Δ/Δ ^(light fill) data overlaid. Genes with gene ontology (GO) annotations in BMP/WNT signaling, or in other growth factor signaling, are indicated by red/green (WT/*Art*^Δ/Δ^) or blue/orange (WT/*Art*^Δ/Δ^) shading, respectively. **(c-e) **RFC data for genes with GO annotations for stress response **(c)**, cell proliferation **(d)**, or cell differentiation **(e) **are shown. RFC for each gene in WT (gray bars) and *Art*^Δ/Δ ^(open bars) samples are overlaid. Individual gene names are indicated. Genes with in BMP, WNT or growth factor signaling pathways are highlighted in red.

To identify genes that may specifically relate to this muted stress response in MSCs, the difference in WT versus *Art*^Δ/Δ ^RFCs (ΔRFC) were determined for each gene, and all genes were then ranked. Using a lenient twofold or greater ΔRFC cutoff, this analysis defined two subsets of differentially regulated genes: those with higher overall expression in WT than in *Art*^Δ/Δ^-positive (ΔRFC in Figure 8a) and those with lower overall expression in WT than in *Art*^Δ/Δ ^(negative ΔRFC in Figure 8a). Analysis of gene ontology (GO) annotations revealed that these differentially perturbed subsets were enriched for genes involved in (1) bone morphogenesic protein (BMP) or Wingless and Int (WNT) signaling pathways (indicated by red/green bars in Figure 8b) and (2) growth factor response and signaling (indicated by blue/orange bars in Figure 8b). These results are interesting, as numerous studies have previously implicated BMP signaling and WNT signaling in multiple types of sarcomagenesis and sarcoma metastasis [46-66,73]. Moreover, alteration in normal growth factor responsiveness is a general hallmark of tumorigenesis in numerous cell types. Altogether, these data reinforce the interpretation that *Art*^Δ/Δ ^MSCs adopt a stress-resistant, aberrantly proliferative behavior likely related to misregulation of normal MSC growth and differentiation pathways.

GO analysis also showed that the list of 157 deregulated genes was enriched for genes annotated to the biological processes of stress response, cell proliferation and cell differentiation. Collectively, these data suggest a model in which cell stress (here via serum withdrawal) normally prompts deregulation of cell proliferation and BMP/WNT-dependent MSC differentiation pathways. We speculate that simultaneous proliferative and antiproliferative signals, evoked by cell stress, culminate in cell death, and that *Art *functions in part to modulate the response to these signals.

## Conclusions

Genomic instability is recognized as a major feature of many, if not all, cancers. However, the mechanisms that maintain normal genomic integrity and their roles in preventing neoplastic transformation are not completely understood. Here we have investigated the role of the nonhomologous end joining pathway of DNA double-stranded break repair in multipotent MSCs/progenitor cells in relation to sarcomagenesis. The cancer stem cell hypothesis posits that stem or stemlike cells are responsible for cancer initiation, metastasis, therapy resistance and relapse after remission. In this context, there is growing evidence to suggest a role for MSCs or MMSs in the development of many sarcomas. Previous studies have shown that *Art*^Δ/Δ ^*Trp53*^Δ/+ ^mice are susceptible to tumorigenesis with shorter latency and an altered spectrum relative to *Trp53*^Δ/+ ^mice. We find overall tumor incidence in *Art*^Δ/Δ ^*Trp53*^Δ/+ ^mice similar to prior studies, but observed a higher incidence of sarcomas than seen in at least one prior study [30]. The basis for this difference in tumor spectrum is not known but may be related to differences in mouse strain background or the prolonged observation period in our study [30]. Importantly, we find evidence for sarcomagenesis without clonal chromosomal translocation in *Art*^Δ/Δ ^cells [67]. This is striking, given the well-documented DNA double-stranded break repair and genome stability functions of *Art. *Rather, we propose that defects in proliferation control following cellular stress can render *Art-*defective (and perhaps other NHEJ-deficient) MSCs or MMSs preneoplastic. In this context, checkpoint regulatory activities of ARTEMIS may be more important than the DSB repair function with regard to sarcoma suppression [12-14]. Taken together, our results suggest that in a sensitized genetic context or with the right series of subsequent genetic hits, potentially preneoplastic MSCs might give rise to sarcomas with differentiation into various lineages [67-69]. It will be interesting to determine, via structure-function studies, which molecular activities of ARTEMIS may differentially contribute to its lymphoma versus sarcoma suppressive functions. It will also be important to assess whether ARTEMIS is relevant to tumor suppression in other tissues, and if so, which functions are important.

In primary multipotent mesenchymal stem or stromal cells (MSC/MMS) we have shown roles for *Art *in both genome stability and cell proliferation control. Together these results suggest that *Art *may function in general DNA double-stranded break repair, as it does in other cell types. But in MSCs, *Art *may also integrate cell cycle responses to cellular stress. Whereas *Art *deficiency did not lead to overt defects in either the number or differentiation function of primary bone marrow-derived MSCs, lack of *Art *did result in aberrant proliferative responses to cellular stress conditions such as ionizing radiation or serum deprivation, conditions that are normally cytostatic to WT MSCs and MMSs. Our data are consistent with a growing body of evidence that ARTEMIS regulates checkpoint responses, perhaps in multiple phases of the cell cycle [11-14]. ARTEMIS is known to be a phosphorylation target of ATR and ATM kinases and was found to be important for proper recovery from both S- and G2/M checkpoints [12,14]. Our data build on the previous studies of *Art*-dependent cell cycle checkpoint control in various cell types [12-14]. We now report a role for *Art *in cell cycle response to IR in primary MSCs. Our control data in primary fibroblasts differ somewhat from previous studies. Geng *et al*. [13] showed that wild-type human embryonic kidney (HEK)-293 cell line cultures experienced a reduced phospho-H3 staining by 6 hours after 3 Gy ionizing irradiation and began showing a rebound in phospho-H3 (and thus mitotic) cells by 12 hours after irradiation [13]. These findings likely reflect an initial G2/M arrest in response to DNA damage, followed by recovery from arrest beginning at or before 12 hours. We found that wild-type primary MEFs experience a modest increase in phospho-H3 as early as 6 hours after 1 Gy of ionizing irradiation, and a significant increase by 12 hours. Possible explanations for the differences between our control MEF data and prior accounts may be differences in cell cycle responses in MEF versus HEK-293 cells, or could reflect kinetic differences that result from different IR doses than those described here [13]. In either case, our overall results are consistent with previous studies, showing an important role for *Art *in modulating cell cycle checkpoint responses to IR. Moreover, our data may suggest a slightly different checkpoint function for *Art *in MSCs, where it appears to enforce, rather than overcome, the G2/M arrest, such that *Art*-defective MSCs remain inappropriately proliferative after IR exposure. Overall, the data presented here indicate a key cell cycle regulatory function for *Art *that may also be cell-context dependent [11-14,70].

In this latter context, we find misregulation of critical growth regulatory pathways in serum-starved *Art*^Δ/Δ ^MSCs. It is not currently known whether the genes we have identified as differentially regulated in serum-starved WT versus *Art*^Δ/Δ ^MSCs directly contribute to the differences in stress sensitivity or alternatively represent biomarkers of the overall differential stress response. However, it is possible that the pathways identified by our differential gene expression analysis influence the exact checkpoint functions of ARTEMIS, perhaps in a cell type-dependent fashion, and may thus account for the phenotypic differences we observe in *Art*^Δ/Δ ^MSCs versus other cell types [12-14]. There is an accumulating literature implicating differentiation and growth factor pathways as critical in normal MSC function and homeostasis. Here we have identified BMP, WNT, and growth factor signaling pathways as differentially affected in serum-starved WT versus *Art*^Δ/Δ ^MSCs. In this context, our identification of altered BMP4 expression is intriguing in light of a recent report showing that BMP2 and BMP4 can induce cytoskeletal changes that modulate cellular differentiation via alterations of cell morphology [71]. Another recent report has suggested that Igfbp5, which we have also identified in this study, may be a key modulator of senescence in some cellular contexts [72]. While we cannot presently rule out other models, our findings may indicate that stress-responsive changes in MSC gene expression are linked to cell cycle control, perhaps via ARTEMIS. Given the excitement that surrounds mesenchymal stem cells and their potential in tissue bioengineering applications, it will be critical to understand the pathways that are important for their normal functioning and for preventing their neoplastic transformation. The findings presented here imply that *Art *encodes a critical modulator of MSC cellular stress and that the cell cycle modulatory function of *Art *represents a key determinant of tumorigenesis arising within tissues engineered from MSCs.

## Methods

### Mice

*Art*^Δ/Δ ^and *Trp53*^Δ/+ ^mice were derived and maintained as previously described [8,26,29]. All animals were maintained in a barrier facility in accordance with Institutional Animal Care and Use Committee-approved protocols.

### Multipotent stromal cell isolation and culture conditions

Total bone marrow was isolated from pools of three or four wild-type C57B6/J or *Art*-null mice between 6 and 10 weeks of age. Independent biological replicates were prepared from pools of independent mice from the appropriate genotypes. Total bone marrow from each pool was plated onto 1- to 150-mm tissue culture plates in 25-mL volume growth medium (α-MEM containing 10% fetal bovine serum (FBS), penicillin/streptomycin, and L-glutamate). When adherent cells became ~80% confluent (approximately day 4-6 of culture), nonadherent cells were washed away and cells were passaged onto 3- to 150-mm culture dishes and then immediately used to initiate experiments or were cryopreserved. For all experiments, only low-passage MSC preparations were used.

### Cytogenetic analysis

To prepare metaphase chromosome spreads, 40 ng/mL colcemid (KaryoMax; Invitrogen, Carlsbad, CA, USA) was added to subconfluent culturing cells to induce metaphase arrest. Cells were then transferred to hypotonic KCl solution (75 mM) for 15 min at 37°C and fixed by two changes of cold 3:1 methanol-acetic acid. Metaphase chromosome preparations were dropped onto slides and further processed for spectral karyotyping (SKY) according to the manufacturer's protocols (Applied Spectral Imaging, Corona, CA, USA). SKY imaging was performed using an ASI complete cytogenetics station (Applied Spectral Imaging) and analyzed with dedicated analysis software (Applied Spectral Imaging).

### Differentiation assays

For osteocyte and adipocyte differentiation, MSCs were plated at 1 × 10^5 ^cells/35-mm well in triplicate. Twenty-four hours after plating, cultures were changed to either osteocyte-specific differentiation medium containing 10 nM dexamethasone (Sigma, St. Louis, MO, USA), 20 mM β-glycerol phosphate (Sigma), and 50 μM L-ascorbic acid (Sigma) or to adipocyte-specific differentiation medium containing 0.5 μM dexamethasone (Sigma), 0.5 μM isobutylmethylxanthine (Sigma), and 50 μM indomethacin (Sigma). Cells were treated with cell-specific differentiation medium for 7-14 days. On the final day of treatment, cells were fixed in 3% formaldehyde/2% sucrose for 10 minutes at room temperature and stained for osteocyte differentiation with alizarin red, which binds to mineralized bone or for adipocyte differentiation with LipidTOX™ (Invitrogen), which binds to neutral lipids. Two wells of each treatment were stained for the cell type generated by differentiation medium, while the third well was stained for the opposite treatment as a specificity control.

### Cell irradiation

MSCs or control fibroblasts were irradiated with the indicated doses of γ-irradiation from a ^137^Cs source, plated in triplicate (0.5 × 10^5 ^cells/35-mm well for MSCs; 0.5 × 10^5 ^cells/60-mm plate for fibroblasts) and cultured for 7 days. Cells were then trypsinized and scraped to dissociate all adherent cells, stained with trypan blue, and counted using a hemacytometer. For immunofluorescence following irradiation, MSCs were cultured on gelatin-coated glass coverslips, irradiated at the indicated dose (ranging from 0-5 Gy), allowed to recover in culture and then fixed and processed for immunofluorescent staining (below in Immunofluorescence methods.).

### Clonogenic Assay

Primary MSCs or MEFs were γ-irradiated at doses from 0 to 3.5 Gy with a ^137^Cs source. A total of 1 × 10^5 ^MEFs or 5 × 10^5 ^cells were plated onto 100-mm culture dishes and cultured until colony formation was visibly obvious for the unirradiated WT control cells. All cells were then fixed in 3% formaldehyde/2% sucrose for 10 minutes at room temperature and permeabilized with 0.5% Triton-X 100 in 1× phosphate-buffered saline (PBS) for 5 minutes at room temperature. Cells were stained in 0.5% crystal violet for 10 minutes at room temperature, and colonies were manually counted. Images were recorded by digital plate scanning.

### Immunofluorescence

MSCs were plated at 1 × 10^5 ^cells/35-mm well in six-well plates containing gelatinized coverslips. Cells treated with regular MSC medium were fixed in 3% formaldehyde/2% sucrose for 10 minutes at room temperature when approximately 80% confluent. Cells treated with osteocyte or adipocyte differentiation medium were fixed in the same manner on days 3, 7 and 14 of treatment. Fixed cells were permeabilized with 0.1% Triton X-100 in 1× phosphate-buffered saline for 15 minutes, blocked in 2% fetal bovine serum for 1 hour at room temperature, and stained with primary antibody to either phospho-H3 (1:200; Upstate; Millipore, Billerica, MA, USA) or phospho-H2AX (1:400; Bethyl, Montgomery, TX, USA) for 16 to 18 hours at 4°C. Cells were then incubated for 30 minutes at room temperature in FITC-labeled goat anti-rabbit IgG (Vector Laboratories, Burlingame, CA, USA) and mounted with Vectashield mounting medium containing 4',6'-diamidino-2-phenylindole counterstain (DAPI; Vector Laboratories, Burlingame, CA). Images were captured by epifluorescence wide-field imaging on a Nikon 90i upright microscope (Nikon, Melville, NY, USA). Images were analyzed using IPLab software (BD Biosciences, Rockville, MD, USA) with minimal image processing.

### Serum starvation

MSCs were plated in triplicate at a density of 0.5 × 10^5 ^cells/35-mm well. After 24 hours, cells were rinsed with PBS and transferred to standard medium with 10% FBS or to medium without serum. Cells were trypsinized, stained with trypan blue and counted on day 7 of treatment.

### Gene Expression Analysis

For gene expression profiling, freshly isolated WT or *Art*^D/D ^MSCs were cultured in duplicate experiments. When cultures reached approximately 80% confluence in 15-cm culture dishes, medium was replaced with fresh basic MSC culture medium (see above in Multipotent stromal cell isolation and culturing methods) either with 10% FBS or lacking serum. Cells were incubated at 37°C in a humidified culture incubator with 5% CO_2 _for 24 hours. Cells were harvested by manual plate scraping, washed once in cold PBS, and stored at -20°C in RNALater (Ambion; Applied Biosciences, Austin, TX, USA) prior to RNA extraction. Standard Affymetrix protocols for Genechip Mouse 430 2.0 (Affymetrix, Santa Clara, CA, USA) were followed to isolate RNA and generate all microarray data.

## List of Abbreviations

DSB: DNA double-stranded break; ESC: embryonic stem cell; Gy: Gray (= 100 rad); IR: ionizing radiation; MSC: mesenchymal stem cell; NHEJ: nonhomologous end joining; RFC: relative fold change; SKY: spectral karyotyping; WT: wild type.

## Authors' contributions

SAM designed and carried out experiments, analyzed data and participated in manuscript preparation. NMD carried out MSC cell culture, assisted in experimental design, prepared samples for microarray analysis and helped with manuscript preparation. KT carried out MSC serum starvation experiments, developed MSC protocols for this study and assisted in manuscript preparation. OF carried out pathology analysis for mouse sarcomas and aided in figure preparation. KDM conceived the study, designed experiments, analyzed results, participated in SKY analysis of sarcomas and MSCs and contributed to manuscript preparation.

## Supplementary Material

Additional file 1 **Aneuploidy without translocations in *Art*-null sarcomas**. Spectral karyotype (SKY) analysis of *Art-*null sarcomas: **(a) **AP812, osteosarcoma; and **(b) **APJ4631, rhabdomyosarcoma. Shown for each is the 4',6'-diamidino-2-phenylindole (DAPI)-stained metaphase (inverted image, top left) with superimposed chromosome contours (blue), spectral image of SKY painted metaphase spread (top, middle), and computer classified image (top, right), as well as the karyotype table showing aneuploidy (bottom). Click here for file

Additional file 2 **The *Art*/*Dclre1c*, encoding ARTEMIS, is transcribed in mesenchymal stem cells (MSCs)**. **(a) **Schematic of reverse transcriptase polymerase chain reaction (RT-PCR) strategy to detect *Art *transcript in wild-type (WT) versus *Art*^Δ/Δ ^MSCs. PCR product detecting exons 1-4 (*Art *ex1-4) is common to both the WT and *Art*^Δ/Δ ^alleles (because the knockout allele eliminates exons 5-6. PCR product detecting exons 1-5 (*Art *ex1-5) is only amplified from WT cells, but not *Art*^Δ/Δ ^cells. **(b) **RT-PCR reactions detecting *Art *ex 1-4, *Art *ex 1-5, or glyceraldehyde 3-phosphate dehydrogenase (GAPDH) (control) transcripts as indicated. Shown are data for either WT or *Art*^Δ/Δ ^fibroblasts or MSCs (as indicated beneath). These data confirm detection of *Art *ex 1-4 in both WT and *Art*^Δ/Δ ^MSCs, but detection of *Art *1-5 only in WT MSCs. This confirms transcriptional expression of *Art *in MSCs and verifies the expected knockout in MSCs from *Art*^Δ/Δ ^mice. Click here for file

Additional file 3 **Control for differentiation specificity of WT or *Art*^Δ/Δ ^MSCs**. **(a) **Fixed *Art*^Δ/Δ ^and *Art *MSCs treated with adipocyte- and osteocyte-specific differentiation medium were stained with the fluorescent lipid binding dye LipidTOX. Cells grown in osteocyte-specific medium are not positive for LipidTOX staining, indicating the absence of adipocytes in these culture conditions. **(b) **Fixed *Art*^Δ/Δ ^and *Art *MSCs treated with adipocyte- and osteocyte-specific differentiation medium were stained with the mineralized bone-specific stain alizarin red. Cells treated with adipogenic medium do not stain with alizarin red, indicating that mineralized bone is not present in adipogenic-treated cells. Click here for file

Additional file 4 **Photomicrograph of WT MSC culture following 2 days of serum withdrawal**. Click here for file

Additional file 5 **Photomicrograph of WT MSC culture following 6 days of serum withdrawal**. Click here for file

Additional file 6 **Photomicrograph of *Art*-null MSC culture following 2 days of serum withdrawal**. Click here for file

Additional file 7 **Photomicrograph of *Art*-null MSC culture following 6 days of serum withdrawal**. Click here for file
