# Supplementary figures and images for: ARTEMIS stabilizes the genome and modulates proliferative responses in multipotent mesenchymal cells
Source: BMC Biol. 2010 Oct 27;8:132. doi: 10.1186/1741-7007-8-132 (PMC2984387; doi:10.1186/1741-7007-8-132)

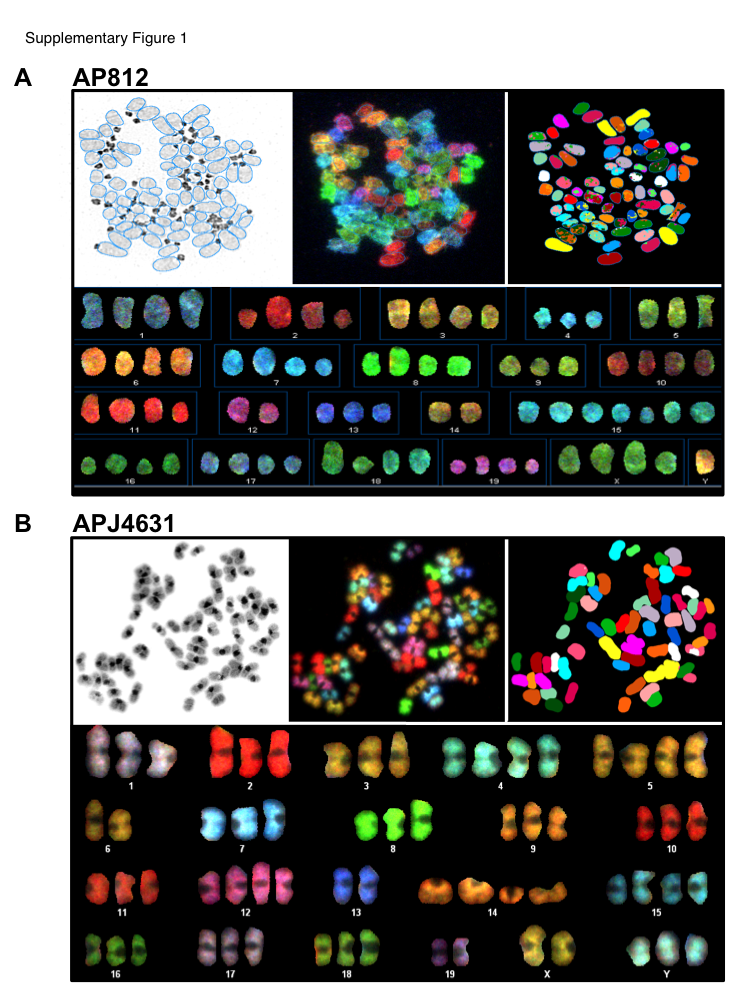

Supplement: Additional file 1 — Aneuploidy without translocations in Art-null sarcomas. Spectral karyotype (SKY) analysis of Art-null sarcomas: (a) AP812, osteosarcoma; and (b) APJ4631, rhabdomyosarcoma. Shown for each is the 4',6'-diamidino-2-phenylindole (DAPI)-stained metaphase (inverted image, top left) with superimposed chromosome contours (blue), spectral image of SKY painted metaphase spread (top, middle), and computer classified image (top, right), as well as the karyotype table showing aneuploidy (bottom). [file 1741-7007-8-132-S1.TIFF]

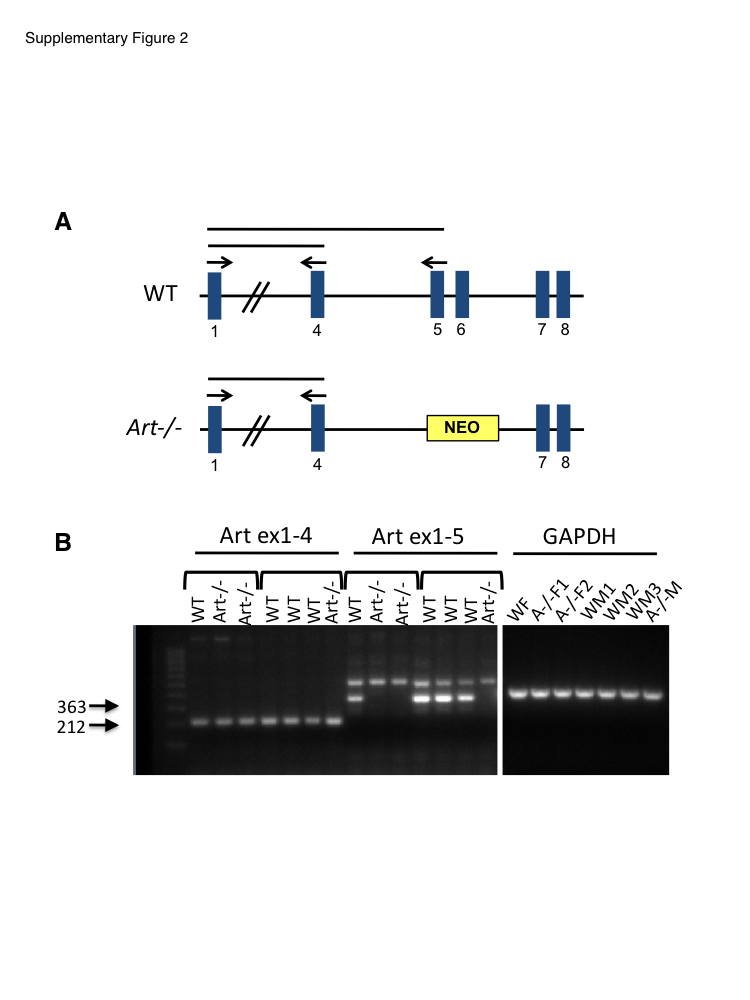

Supplement: Additional file 2 — The Art/Dclre1c, encoding ARTEMIS, is transcribed in mesenchymal stem cells (MSCs). (a) Schematic of reverse transcriptase polymerase chain reaction (RT-PCR) strategy to detect Art transcript in wild-type (WT) versus ArtΔ/Δ MSCs. PCR product detecting exons 1-4 (Art ex1-4) is common to both the WT and ArtΔ/Δ alleles (because the knockout allele eliminates exons 5-6. PCR product detecting exons 1-5 (Art ex1-5) is only amplified from WT cells, but not ArtΔ/Δ cells. (b) RT-PCR reactions detecting Art ex 1-4, Art ex 1-5, or glyceraldehyde 3-phosphate dehydrogenase (GAPDH) (control) transcripts as indicated. Shown are data for either WT or ArtΔ/Δ fibroblasts or MSCs (as indicated beneath). These data confirm detection of Art ex 1-4 in both WT and ArtΔ/Δ MSCs, but detection of Art 1-5 only in WT MSCs. This confirms transcriptional expression of Art in MSCs and verifies the expected knockout in MSCs from ArtΔ/Δ mice. [file 1741-7007-8-132-S2.TIFF]

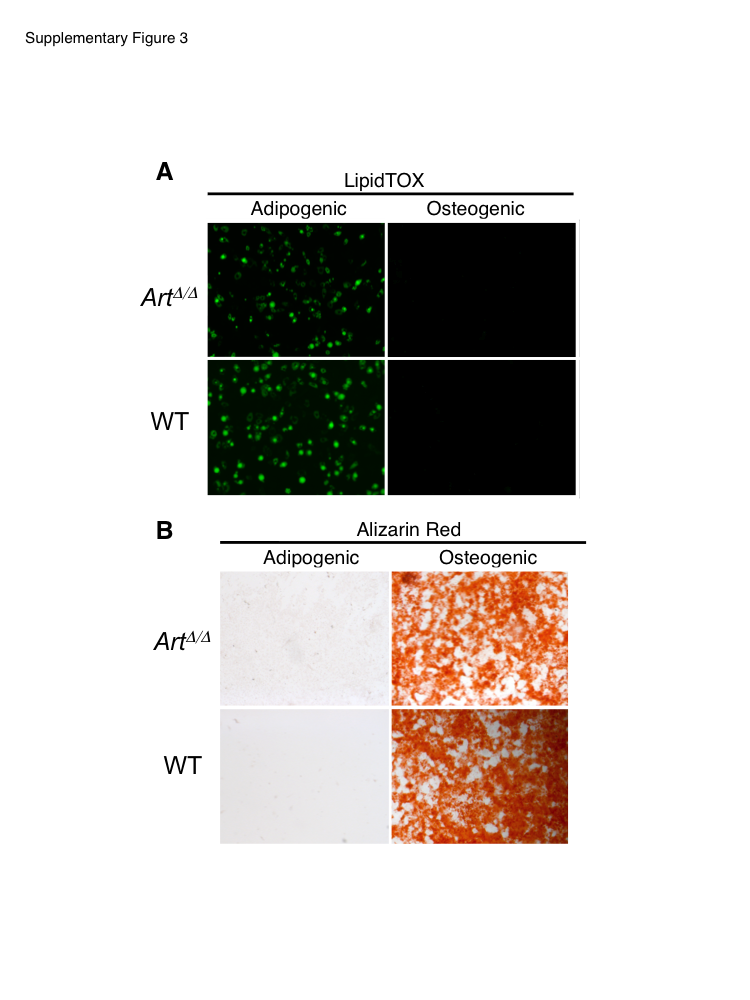

Supplement: Additional file 3 — Control for differentiation specificity of WT or ArtΔ/Δ MSCs. (a) Fixed ArtΔ/Δ and Art MSCs treated with adipocyte- and osteocyte-specific differentiation medium were stained with the fluorescent lipid binding dye LipidTOX. Cells grown in osteocyte-specific medium are not positive for LipidTOX staining, indicating the absence of adipocytes in these culture conditions. (b) Fixed ArtΔ/Δ and Art MSCs treated with adipocyte- and osteocyte-specific differentiation medium were stained with the mineralized bone-specific stain alizarin red. Cells treated with adipogenic medium do not stain with alizarin red, indicating that mineralized bone is not present in adipogenic-treated cells. [file 1741-7007-8-132-S3.TIFF]

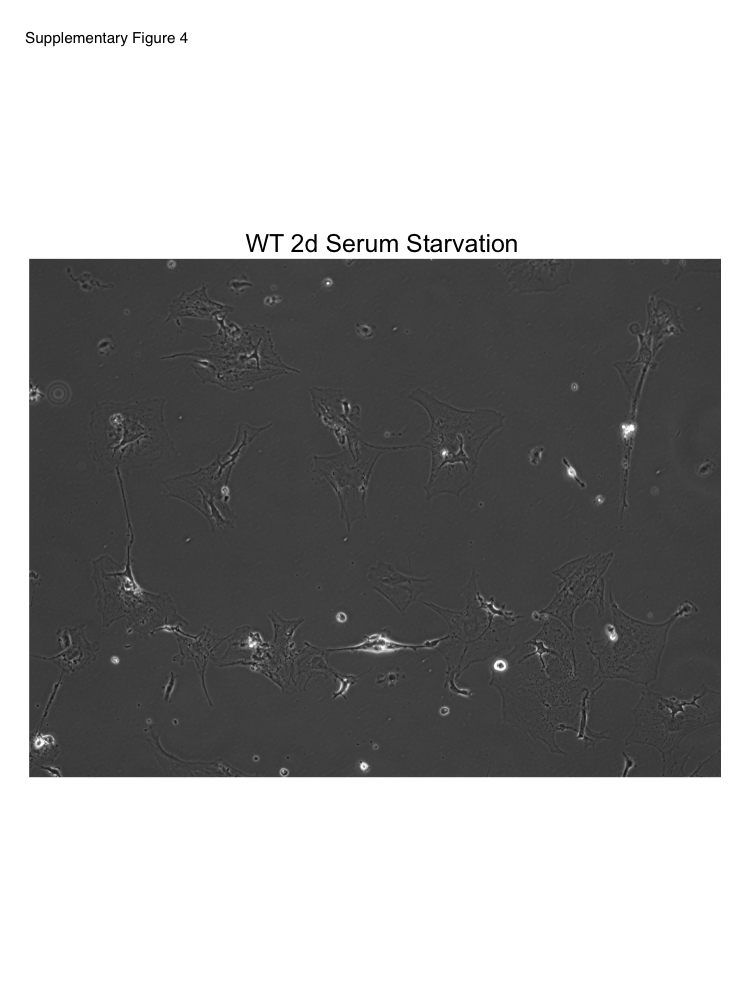

Supplement: Additional file 4 — Photomicrograph of WT MSC culture following 2 days of serum withdrawal. [file 1741-7007-8-132-S4.TIFF]

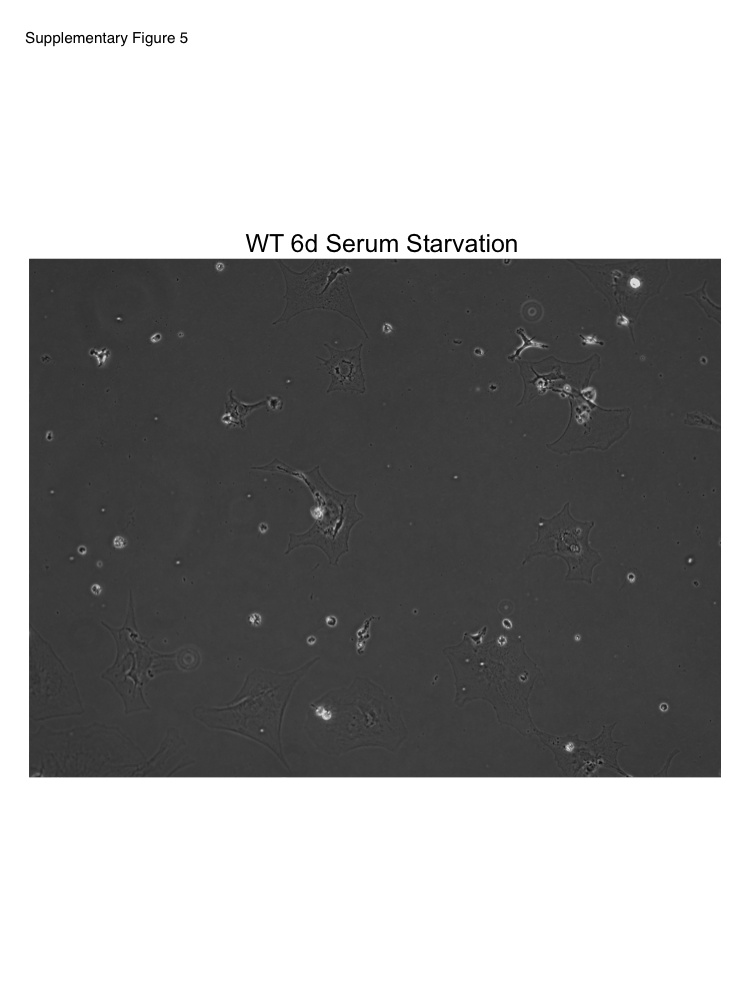

Supplement: Additional file 5 — Photomicrograph of WT MSC culture following 6 days of serum withdrawal. [file 1741-7007-8-132-S5.TIFF]

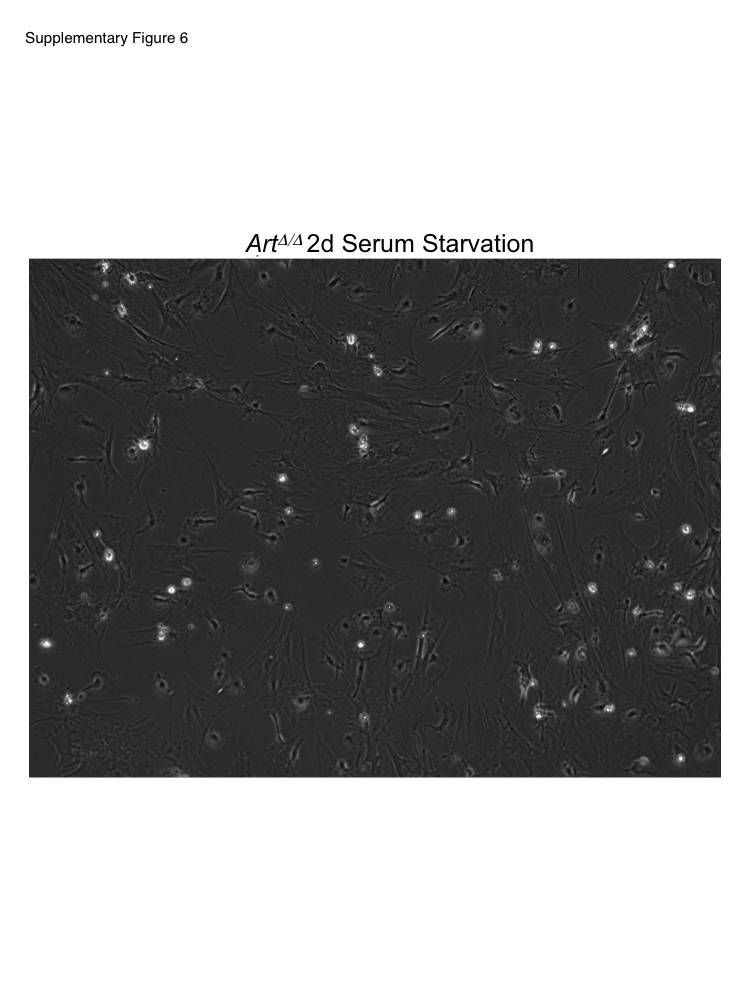

Supplement: Additional file 6 — Photomicrograph of Art-null MSC culture following 2 days of serum withdrawal. [file 1741-7007-8-132-S6.TIFF]

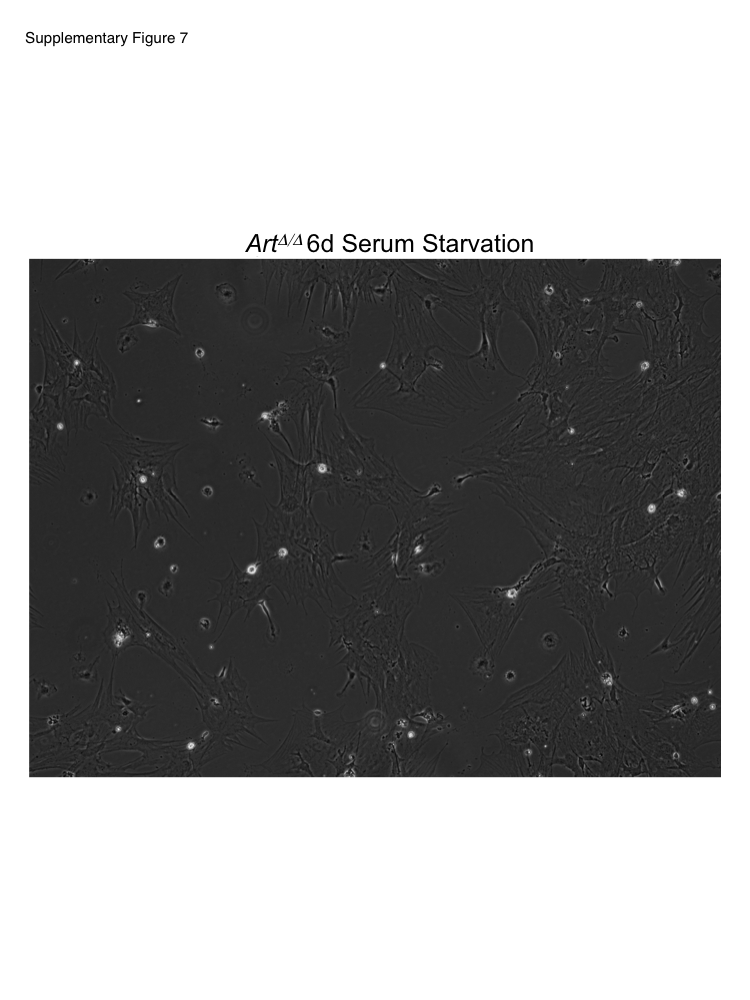

Supplement: Additional file 7 — Photomicrograph of Art-null MSC culture following 6 days of serum withdrawal. [file 1741-7007-8-132-S7.TIFF]
